# Use of a Four-miRNA Panel as a Biomarker for the Diagnosis of Stomach Adenocarcinoma

**DOI:** 10.1155/2020/8880937

**Published:** 2020-11-07

**Authors:** Xuan Chen, Xinji Li, Xiqi Peng, Chunduo Zhang, Kaihao Liu, Guocheng Huang, Yongqing Lai

**Affiliations:** ^1^Guangdong and Shenzhen Key Laboratory of Male Reproductive Medicine and Genetics, Peking University Shenzhen Hospital, Institute of Urology of Shenzhen PKU-HKUST Medical Center, Shenzhen, Guangdong 518036, China; ^2^Shantou University Medical College, Shantou, Guangdong 515041, China; ^3^Anhui Medical University, Hefei, Anhui 230032, China

## Abstract

**Background:**

MicroRNAs (miRNAs) have been applied to cancer diagnosis taking into account their role in tumorigenesis. The main purpose of our study was to confirm the possibility of using miRNAs as noninvasive biomarkers for stomach adenocarcinoma (STAD) diagnosis.

**Methods:**

A total of 246 participants (130 STAD patients and 116 healthy controls (HCs)) were enrolled in this 3-phase study. Five STAD pools and 3 HC pools (with 4 participants in each pool) were used for the screening of the 28 miRNAs using quantitative reverse transcription-polymerase chain reaction (qRT-PCR). The training phase (30 STAD patients vs. 24 HCs) and validation phase (80 STAD patients vs. 80 HCs) were used to further verify the identity of these miRNAs. Kaplan–Meier survival analysis and bioinformatics analysis were also used.

**Results:**

The expression levels of miR-125b-5p and miR-196a-5p were upregulated in STAD serum, compared with the HCs, while miR-1-3p and miR-149-5p showed the opposite result. A four-serum miRNA panel was constructed, and the area under the receiver operating characteristic curve (AUC) was found to be 0.892 (95% CI: 0.834 to 0.936, sensitivity = 86.25%, specificity = 78.75%). Only miR-125b-5p expression showed a significant difference between STAD patients and NCs in the survival analysis. The neurotrophin signaling pathway was associated with 4 miRNAs identified in STAD patients.

**Conclusion:**

The four-serum miRNA panel has great potential to be used as a noninvasive biomarker for STAD diagnosis.

## 1. Introduction

Gastric cancer has been ranked third for the number of cancer-associated deaths globally. 1.2 million new cases of stomach cancer and 834,000 deaths were reported worldwide in 2016 [1]. However, due to a lack of typical symptoms, the diagnosis of most patients is usually delayed and advanced stage patients have already lost the opportunity for surgery at diagnosis. Based on the survey by the American Cancer Society, the trend in the 5-year relative survival rate of gastric cancer has been gradually rising but remains at a low level, at approximately 29% [2]. Gastric cancer is highly malignant due to its extensive metastatic ability to form metastases in a variety of organs, such as the lymphatic glands, liver, and ovaries [3]. Most malignant gastric cancers (about 95%) are of the adenocarcinoma histological type, which include intestinal and diffuse types that are classified based on the Lauren classification [4]. If the opportunity to undergo surgery is missed, the median survival time of advanced-stage stomach adenocarcinoma (STAD) is only 9-10 months [5]. Therefore, concentrating on the early diagnosis of STAD could greatly improve the overall survival rate of gastric cancer patients [6]. Currently, the main diagnostic method used is endoscopy or surgical biopsy, which examined the gold standard for diagnosing STAD. Some countries have benefited by using strategies that can be used to screen (endoscopy) for a large population or high-risk individuals. However, in some countries, this strategy can be difficult to implement due to the high cost, inconvenience, and invasiveness [7]. Hence, novel and convenient diagnostic measures need to be identified to enhance STAD diagnostic methods. During recent years, noninvasive biomarkers have been increasingly used for clinical examination. Some studies have also reported on the use of liquid biopsy, indicating that body fluids, such as urine and sperm, contain enormous potential to be used for the diagnosis of cancer and other diseases [8, 9]. These studies all involve the use of miRNAs.

The potential of using noninvasive biomarkers, such as microRNAs (miRNAs), for the diagnosis of diseases, including cancer, has been explored [10]. Although miRNAs contain only 20-25 nucleotides, they play a crucial role in cell biology, including in processes such as proliferation, apoptosis, and invasion [11]. miRNAs not only regulate relative genes during the cell process but also, more importantly, may serve as indicators in body fluids, such as blood and urine [12]. Based on the characteristics of miRNAs, specific miRNAs may be suitable for STAD diagnosis.

A novel study extracted miRNAs from urine to establish a compound panel for the detection of gastric cancer [8]. In our study, we used 246 serum samples and set up 3 phases to explore the potential ability of miRNAs to be used for STAD diagnosis. Quantitative reverse transcription-polymerase chain reaction (qRT-PCR), which is mainly based on the number of nucleic acid targets and the increase in the duration of fluorescence and is considered the gold standard method for the identification of miRNAs [13], was used to create a composite miRNA panel. Based on the receiver operating characteristic (ROC) curve analysis, we evaluated the diagnostic efficiency. Additionally, survival and bioinformatics analyses were also conducted.

## 2. Materials and Methods

### 2.1. Sample Gathering

Every procedure included in this study was undertaken with the approval of the Ethics Committee of Shenzhen Hospital, Peking University. Written informed consent was obtained from all participants. All serum samples were collected from November 2017 to August 2019 and were obtained from 130 STAD patients and 116 healthy controls (HCs). The patients were diagnosed as STAD based on their histology, without any treatment administer prior to specimen collection. The HCs were free of interference factors, such as other conflicting diseases or a history of cancer. The clinical features of all participants are illustrated in Table 1.

### 2.2. Research Process

First, 28 miRNAs associated with STAD were chosen from the PubMed and the Gene Expression Omnibus databases using the following search strategy: (“Stomach Neoplasms”[Mesh] OR (gastric cancer)) AND (“MicroRNAs”[Mesh] OR miRNA OR microRNA). Accordingly, 3 sequential phases were implemented (Figure S1). During the screening phase, 20 STAD patients and 12 HCs were chosen at random to create 5 STAD pools and 3 HC pools (each pool contained 4 serum samples). For the purpose of evaluating the expression levels of the 28 miRNAs, we applied high throughput qRT-PCR to distinguish aberrantly expressed miRNAs between the two groups with the cut-off criteria *p* value of <0.05 and fold change (FC) of >1.5 or <–1.5. Next, in the training phase, we randomly selected 30 new STAD samples and 24 HC samples to further verify the differential expression of the 12 candidate miRNAs using qRT-PCR. Eight miRNAs that were still differentially expressed (*p* value < 0.05) were selected for further analysis. During the validation phase, the expression levels of these 8 miRNAs were confirmed through the rest of the STAD and HCs. Finally, 7 miRNAs with differential expression (*p* value < 0.05) and of diagnostic value (using receiver operating characteristic curves analysis) were included in the construction of a diagnostic miRNA panel for STAD.

### 2.3. Sample Handling and RNA Extraction

10 ml of peripheral blood was obtained from all participants who did not receive any treatment, centrifuged, and disposed within 2 hours. Centrifugation was first conducted at 1000 g for 10 minutes and then at 15,000 g for 5 minutes at 4°C. After adding 2 *μ*l of synthetic C. elegans, miR-39 (cel-miR-39) (10 nM/l; RiboBio, Guangzhou, China) was used as an external reference and a normalization tool. Every serum specimen was extracted and purified. For RNA extraction, a TRIzol LS isolation kit (Thermo Fisher Scientific, Waltham, MA, USA) was used as specified in its operating manual. Then, the extracted RNA was dissolved in 30 *μ*l of RNase-free water and stored at -80°C for further use. RNA concentration and purity were determined using a NanoDrop 2000 spectrophotometer (NanoDrop, Wilmington, DE, USA).

### 2.4. Quantitative Reverse Transcription-Polymerase Chain Process

After specific primers of reverse transcription obtained using the Bulge-Loop miRNA qRT-PCR Primer Set (RiboBio, Guangzhou, China) were added to the extracted RNA, the amplification of miRNAs was performed in a LightCycler 480 Real-Time PCR System (Roche Diagnostics, Mannheim, Germany) using a SYBR Green qPCR kit (SYBR Pre-mix Ex Taq II, TaKaRa). Real-time polymerase chain reaction was performed in 384-well plates, first at 95°C for 30s, and then 30 cycles of 95°C for 10 s, 60°C for 20 s, and 70°C for 10 s. Finally, the specificity of the PCR products was determined through melting curve analysis. To ensure the accuracy of the results, the experiment was repeated at least 3 times. The 2^−△△Cq^ method was used to determine the relative expression levels of the target miRNAs [14].

### 2.5. Survival Analysis

Kaplan–Meier survival analysis and the logrank test were used to predict the survival rate in OncoLnc (http://www.oncolnc.org/). We downloaded data on 400 STAD patients from TCGA database and divided them into a high-expression level group and a low-expression level group, based on the expression levels of the target miRNAs (high limit: 60% and low limit: 40%). We analyzed the survival data of the 4 miRNAs using TCGA database.

### 2.6. Bioinformatics Analysis

MiRWalk3.0 (http://mirwalk.umm.uni-heidelberg.de/), a comprehensive database on miRNA-target interactions, was employed to predict the target genes of the candidate miRNAs involved in STAD. In addition, the predicted target genes were added into the DAVID database (version 6.8) (http://david.abcc.ncifcrf.gov/) for Gene Ontology (GO) annotation and Kyoto encyclopedia of genes and genomes (KEGG) pathway analysis. GO analysis included biological processes (BP), cellular components (CC), and molecular functions (MF).

### 2.7. Statistical Analysis

We used count percentages or the mean ± standard deviation (if the numbers were continuous) to express the demographic and clinical characteristics between different groups. For the statistical analysis, different data corresponding to the different methods, including multiple comparisons among separate independent phases, were determined using the Kruskal-Wallis rank test, and the differential expression levels of each miRNA between the STAD patients and the HC samples were determined using the Student's *t*-test or the Mann–Whitney test. Multiple logistic regression analysis was used to construct the miRNA profiles, while the diagnostic capability of the miRNA panel was determined using receiver operating characteristic (ROC) curves and the area under the ROC curve (AUC). We utilized SPSS software (SPSS 23.0 Inc., Chicago, IL), GraphPad Prism 8 (GraphPad Software Inc., La Jolla, CA), and Medcalc (version 19) to conduct the statistical analysis. If the *p* value was lower than 0.05, the result was regarded as being statistically significant.

## 3. Results

### 3.1. Demographics and Clinical Manifestations in the Patients

130 STAD patients and 116 HCs were enrolled in our experiment. There was no statistical significance in age between the STAD group and the HC group (*p* values > 0.05). Detailed information on participants at each phase is listed in Table 1. The information included tumor diameter, lymphatic metastasis, TNM stage differentiation, and invasion depth. Patients were histologically diagnosed with STAD based on the World Health Organization standards. During each phase, we compared STAD patients with HCs who had no history of cancer or interferential diseases.

### 3.2. Candidate miRNAs in the Screening Phase

**Figure 1:**
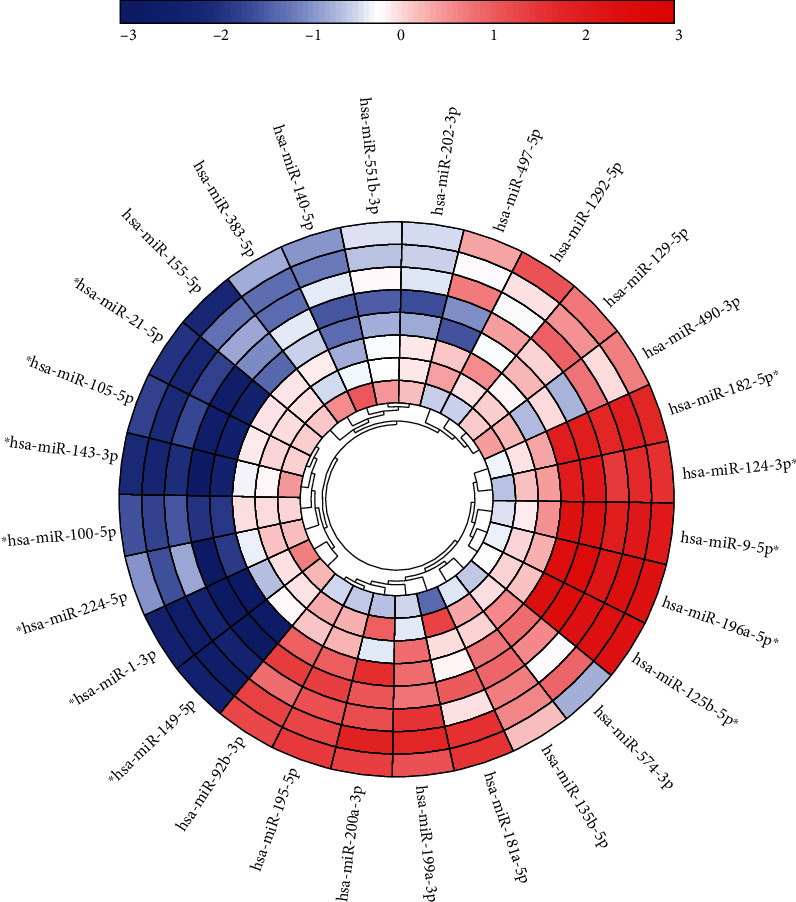
Heatmap of the 28 miRNAs during the preliminary screening using 5 STAD patient pools and 3 HC pools. The three inner circles represent the HC pools. High expression is shown in red and low expression in blue. The 12 miRNAs marked with an asterisk were passed to the next phase (*p* < 0.05 and fold change (FC) > 1.5 or <–1.5).

As shown in Figure 1, first, we screened 28 miRNAs (miR-196a-5p, miR-125b-5p, miR-9-5p, miR-182-5p, miR-124-3p, miR-200a-3p, miR-195-5p, miR-199a-3p, miR-92b-3p, miR-181a-5p, miR-135b-5p, miR-129-5p, miR-574-3p, miR-1292-5p, miR-490-3p, miR-497-5p, miR-551b-3p, miR-202-3p, miR-383-5p, miR-140-5p, miR-155-5p, miR-224-5p, miR-100-5p, miR-105-5p, miR-21-5p, miR-143-3p, miR-149-5p, and miR-1-3p) in the 5 STAD pools and 3 HC pools. Based on the expression level and under a standard fold change (FC) of >1.5 or <–1.5 and a *p* value of <0.05, the 12 miRNAs were selected and moved to the next phase. Among of them, 5 miRNAs (miR-196a-5p, miR-125b-5p, miR-9-5p, miR-182-5p, and miR-124-3p) were overexpressed and the other miRNAs (miR-224-5p, miR-100-5p, miR-105-5p, miR-21-5p, miR-143-3p, miR-149-5p, and miR-1-3p) were downregulated in STAD patients compared with HCs. Table S1 shows the details of the expression levels of the 28 miRNAs in both pools.

### 3.3. Further Confirmation of the Candidate miRNAs in the Training Phase

**Figure 2:**
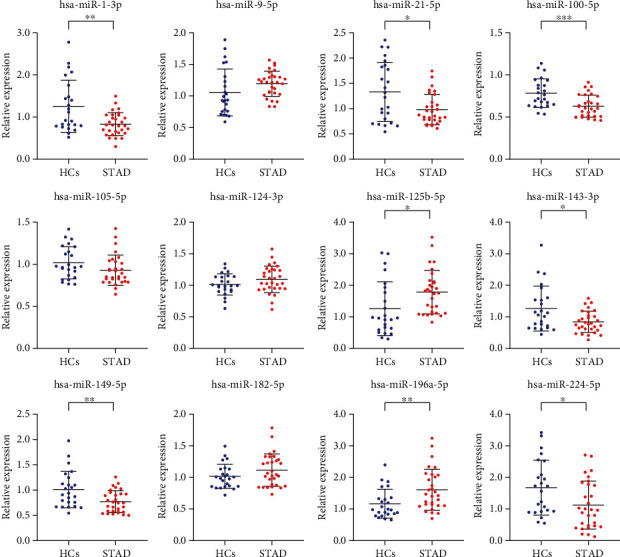
Relative expression levels of the 12 candidate miRNAs during the training phase. This phase included 30 STAD patient serum and 24 HC serum samples. ^∗^*p* < 0.05; ^∗∗^*p* < 0.01; ^∗∗∗^*p* < 0.001.

The training phase was used to further confirm the stability of the expression level differences between STAD patients and HCs of the 12 candidate miRNAs identified through preliminary screening (miR-196a-5p, miR-125b-5p, miR-9-5p, miR-182-5p, miR-124-3p, miR-224-5p, miR-100-5p, miR-105-5p, miR-21-5p, miR-143-3p, miR-149-5p, and miR-1-3p). We performed qRT-PCR analysis on 30 STAD serum samples and 24 HC serum samples. As shown in Figure 2, 8 miRNAs (miR-196a-5p, miR-125b-5p, miR-224-5p, miR-100-5p, miR-21-5p, miR-143-3p, miR-149-5p, and miR-1-3p) (*p* value < 0.05) continued to be aberrantly expressed during the training phase.

### 3.4. Validation of the 8 miRNAs and Their Diagnostic Value

**Figure 3:**
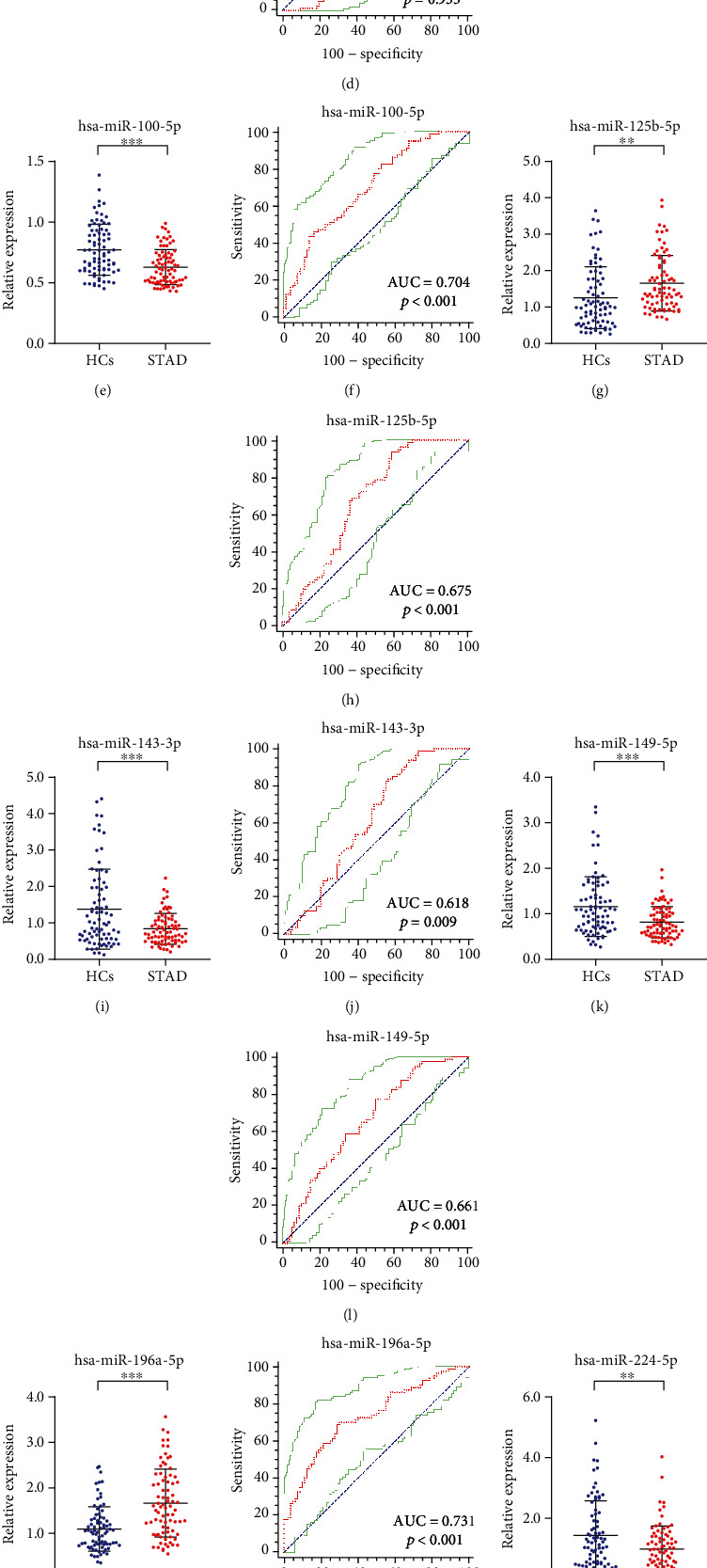
Relative expression levels and receiver operating characteristic curve (ROC) analyses of the 8 selected miRNAs in the validation phase. This phase included 80 STAD patient serum and 80 HC serum samples. The miRNAs that were highly expressed in STAD were (g) miR-125b-5p and (m) miR-196a-5p. Their AUCs were (h) 0.675 (95% CI: 0.596 to 0.747) and (n) 0.731 (95% CI: 0.655 to 0.798), respectively. miRNAs with significantly low-expression levels were (a) miR-1-3p, (e) miR-100-5p, (i) miR-143-3p, (k) miR-149-5p, and (o) miR-224-5p. Their AUCs were (b) 0.719 (95% CI: 0.550 to 0.700) for miR-1-3p, (f) 0.704 (95% CI: 0.627 to 0.773) for miR-100-5p, (j) 0.618 (95% CI: 0.538 to 0.693) for miR-143-3p, (l) 0.661 (95% CI: 0.582 to 0.734) for miR-149-5p, and (p) 0.606 (95% CI: 0.526 to 0.683) for miR-224-5p. The expression level of miR-21-5p made no sense (*p* > 0.05). ^∗^*p* < 0.05; ^∗∗^*p* < 0.01; ^∗∗∗^*p* < 0.001.

The 8 candidate miRNAs included in the validation phase were verified using 80 STAD patients and 80 HCs (Figure 3). Based the results of the validation phase, in addition to miR-21-5p, the expression levels of the other 7 miRNAs (miR-196a-5p, miR-125b-5p, miR-224-5p, miR-100-5p, miR-143-3p, miR-149-5p, and miR-1-3p) continued to be dysregulated (*p* value < 0.05). Compared with the HCs, the expression levels of miR-125b-5p and miR-196a-5p were overexpressed in STAD patients, while those of miR-149-5p, miR-143-3p, miR-224-5p, miR-1-3p, and miR-100-5p showed an opposite result.

Moreover, the ROC curves of the 6 candidate miRNAs were plotted to evaluate their respective diagnostic capabilities. Figure 3 displays their respective AUCs—0.719 (95% confidence interval (CI): 0.643 to 0.787, Figure 3(b)) for miR-1-3p; 0.504 (95% CI: 0.424 to 0.584, Figure 3(d)) for miR-21-5p; 0.704 (95% CI: 0.627 to 0.773, Figure 3(f)) for miR-100-5p; 0.675 (95% CI: 0.596 to 0.747, Figure 3(h)) for miR-125b-5p; 0.618 (95% CI: 0.538 to 0.693, Figure 3(j)) for miR-143-3p; 0.661 (95% CI: 0.582 to 0.734, Figure 3(l)) for miR-149-5p; 0.731 (95% CI: 0.655 to 0.798, Figure 3(n)) for miR-196a-5p; and 0.606 (95% CI: 0.526 to 0.683, Figure 3(p)) for miR-224-5p.

### 3.5. Building up a Compound miRNA Panel for STAD Diagnosis

**Figure 4:**
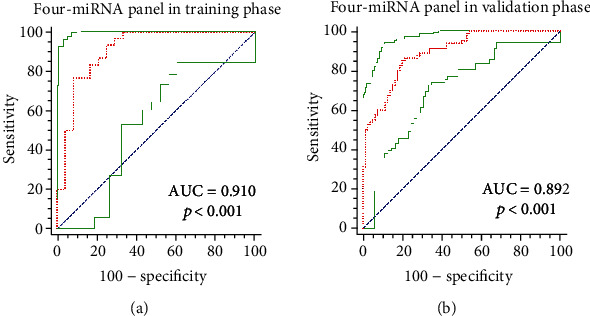
The receiver operating characteristic curve analyses of the 4-miRNA panel in the training and validation phases and their AUCs. The panel was composed of miR-125b-5p, miR-196a-5p, miR-1-3p, and miR-149-5p. Their AUC values were 0.910 (95% CI: 0.800 to 0.971, sensitivity = 93.33%, specificity = 75.00%) in the training phase and 0.892 (95% CI: 0.834 to 0.936, sensitivity = 86.25%, specificity = 78.75%) in the validation phase.

To enhance the sensitivity and specificity of the miRNAs to be used as a diagnostic biomarker, we combined these representative miRNAs together to form a panel of composite miRNAs. Based on the respective multiple logistical regression analytics conducted on the training and validation phases, a panel of 4 miRNAs (miR-125b-5p, miR-196a-5p, miR-1-3p, and miR-149-5p) showed a higher diagnostic efficacy. Hence, the ROC curve and stepwise logistical regression model were constructed for confirmation of the results (Figure 4). Their AUCs were 0.910 (95% CI: 0.800 to 0.971, sensitivity = 93.33%, specificity = 75.00%) in the training phase and 0.892 (95% CI: 0.834 to 0.936, sensitivity = 86.25%, specificity = 78.75%) in the validation phase. The panel was constructed using the formula: Logit(*p*) = 3.038 + 1.241 × miR‐125b‐5p + 2.037 × miR‐196a‐5p‐2.557 × miR‐1 − 3p − 4.581 × miR‐149‐5p in the training phase and Logit(*p*) = 0.468 + 1.126 × miR‐125b‐5p + 2.064 × miR‐196a‐5p − 2.189 × miR‐1‐3p − 3.019 × miR‐149‐5p in the validation phase.

### 3.6. Association between the Relative Expression of the Four Candidate Serum miRNAs and Clinical Features

We further investigated whether the expression levels of the four candidate miRNAs were related with the clinical manifestations using the Wilcoxon-Mann–Whitney test (by including all participants in the training and validation phases). As shown in Table 2, certain clinical features, including tumor diameter, lymphatic metastasis, and differentiation, showed no significant association with the changes in expression levels. For TNM staging, there were differences in the miRNA expression levels between STAD patients and HCs (*p* = 0.015) for miR-125b-2p and miR-1-3p. Additionally, compared with the HCs, the cancer tissue of the STAD patients showed deeper invasion, and the expression levels of miR-1-3p in serum were lower.

### 3.7. Survival and Bioinformatics Analysis of the Four Candidate miRNAs

**Figure 5:**
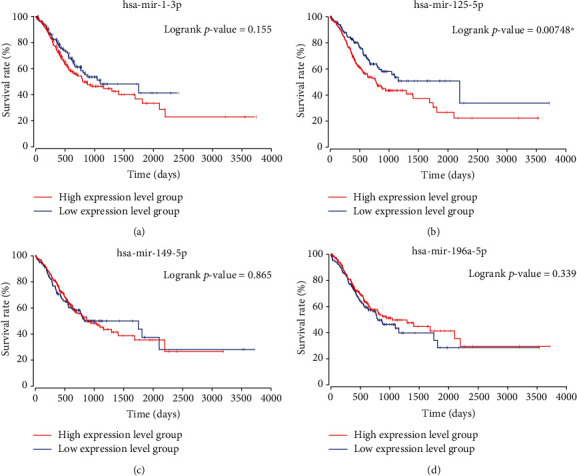
Kaplan–Meier survival analysis and logrank test of the miRNAs included in the panel. Data on 400 STAD patients obtained from TCGA database were included in the analysis. (a) miR-1-3p, logrank *p* value = 0.155. (b) miR-125b-5p, logrank *p* value = 0.00748. (c) miR-149-5p, logrank *p* value = 0.865. (d) miR-196a-5p, logrank *p* value = 0.339. Only miR-125b-5p showed a statistical difference (*p* < 0.05).

Kaplan–Meier survival analysis and logrank test were performed on the data of 400 STAD patients obtained from TCGA dataset. Patients with the top 60% of miRNA expression levels were considered as the high-expression level group and the others were classified as the low-expression level group. As shown by the Kaplan–Meier survival curves (Figure 5), only miR-125b-5p was associated with the survival rate of STAD. The expression level of miR-125b-5p was higher, and the prognosis of STAD was worse.

**Figure 6:**
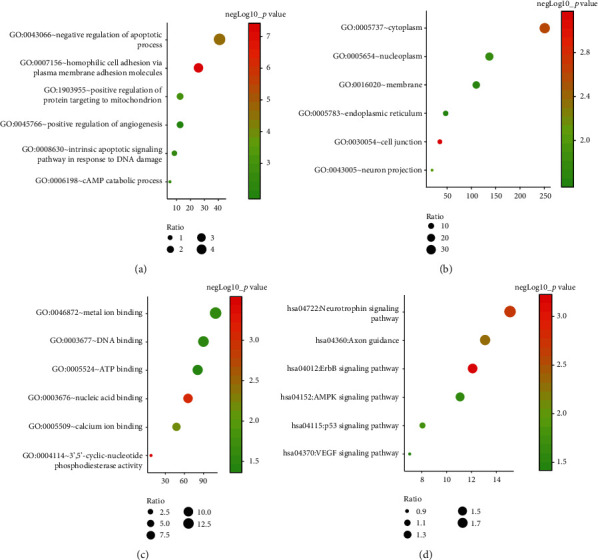
GO functional annotation and KEGG pathway enrichment analysis. The target genes of miR-125b-5p, miR-196a-5p, miR-1-3p, and miR-149-5p were predicted. (a) Biological processes (BPs). (b) Cellular components (CCs). (c) Molecular functions (MFs) in the GO functional annotation. (d) KEGG pathway analysis.

Using miRWalk3.0, if a gene was predicted for more than 2 candidate miRNAs, it was selected as the target gene. A total of 814 genes were predicted. Then, the target genes underwent GO functional annotation and KEGG pathway enrichment analyses using the DAVID database. The top 6 GO enriched terms, including biological process (BP), cellular component (CC), and molecular function (MF) terms, are listed in Figures 6(a)–6(c). BPs (Figure 6(a)) included negative regulation of apoptotic process (GO:0043066), homophilic cell adhesion via plasma membrane adhesion molecules (GO:0007156), positive regulation of protein targeting to mitochondrion (GO:1903955), positive regulation of angiogenesis (GO:0045766), intrinsic apoptotic signaling pathway in response to DNA damage (GO:0008630), and cAMP catabolic process (GO:0006198). The CCs (Figure 6(b)) included cytoplasm (GO:0005737), nucleoplasm (GO:0005654), membrane (GO:0016020), endoplasmic reticulum (GO:0005783), cell junction (GO:0030054), and neuron projection (GO:0043005).  Figure 6(c) shows the MFs, included metal ion binding (GO:0046872), DNA binding (GO:0003677), ATP binding (GO:0005524), nucleic acid binding (GO:0003676), calcium ion binding (GO:0005509), and 3′,5′-cyclic nucleotide phosphodiesterase activity (GO:0004114). The top 6 enriched terms in the KEGG pathway analysis, which are shown in Figure 6(d), included the neurotrophin signaling pathway, Axon guidance, ErbB signaling pathway, AMPK signaling pathway, p53 signaling pathway, and VEGF signaling pathway.

## 4. Discussion

Gastric cancer leads to the third highest tumor mortality rate in the world, with an exceptionally low 5-year survival rate. Among them, STAD accounted for about 95% of all malignant tumors, and its prognosis was poor. The common methods used for the diagnosis of STAD are endoscopy and biopsy, which are invasive, and their large-scale application is challenging. In addition, due to atypical symptoms, STAD is usually diagnosed at the late stages. Therefore, it is necessary to identify noninvasive methods for the diagnosis of STAD. Thus far, most studies have explored the diagnostic value of peripheral miRNAs in many cancers based on the roles of the miRNAs. For example, Zhou et al. clarified that a 6-miRNA signature had the potential to be used to diagnose patients with esophageal squamous cell carcinoma [15]. Moreover, Zhao et al. clarified that a 4-miRNA signature could be used for the diagnosis of gastric cancer [16]. In our study, we focused on the diagnosis of STAD. We aimed to group certain serum miRNAs with a high level of sensitivity and specificity into a noninvasive panel that could be used to enrich STAD diagnostic tools. Based on the experimental framework, 130 STAD patients and 116 HCs were enrolled in our study, which included 3 distinct phases and representative analyses. First, 28 miRNAs were tested using 5 STAD pools and 3 HC pools. Then, the training phase was conducted using data on 30 STAD patients and 24 HCs, while the validation phase was conducted using data on 80 STAD patients and 80 HCs by performing qRT-PCR analyses. Finally, compared with the HCs, 7 miRNAs with abnormal expression in STAD patients, of which 2 (miR-125b-5p and miR-196a-5p) were upregulated and 5 (miR-149-5p, miR-143-3p, miR-224-5p, miR-1-3p, and miR-100-5p) were downregulated, were selected as candidate miRNAs. Moreover, to improve the sensitivity and specificity of detection, a 4-miRNA (miR-125b-5p, miR-196a-5p, miR-1-3p, and miR-149-5p) serum panel was constructed. Based on the AUC analysis, the ROC of the compound panel were 0.910 (95% CI: 0.800 to 0.971, sensitivity = 93.33%, specificity = 75.00%) in the training phase and 0.892 (95% CI: 0.834 to 0.936, sensitivity = 86.25%, specificity = 78.75%) in the validation phase (Figure 4). Its computable formula was Logit(*p*) = 0.468 + 1.126 × miR‐125b‐5p + 2.064 × miR‐196a‐5p − 2.189 × miR‐1‐3p − 3.019 × miR‐149‐5p.

In addition to the novel noninvasive panel, Kaplan–Meier survival analysis was used to predict the survival rate conferred by each identified miRNA. Among the 4 candidate miRNAs, only the aberrant regulation of miR-125b-5p was associated with the survival rate of the STAD patients. Other studies, such as the report by Zhang et al., have reached similar conclusions [17]. The study by Zhang et al. determined the prediction ability of 3 miRNAs (including miR-125b-5p) for STAD survival. Furthermore, in our experiment, miRNA-125b-5p was upregulated and significantly associated with the TMN stage of the STAD patients. During recent years, researchers have explored the potential mechanism by which miRNA-125b (precursor of miRNA-125b-5p) is involved in gastric cancer. One other previous research showed that miR-125b was as an intermediary between KDM4B (a histone-modifying enzyme) and Wnt signaling [18]. Another demonstrated that miR-125b targeted the PPP1CA-Rb signal pathway to enhance the migration and invasion of gastric carcinoma cells [19]. The two studies jointly confirmed that a high-expression level of miR-125b-5p may lead to poor prognosis of gastric cancer. Together, these results indicated that serum miR-125b-5p could be used as a biomarker for STAD diagnosis and prognosis.

Among the 4 candidate miRNAs, miR-196a-5p showed the largest diagnostic value (AUC: 0.731, 95% CI: 0.655 to 0.798; Figure 3(p)). It was found to be a potential biomarker for STAD diagnosis. Particularly, miR-196a-5p was involved in many of the responses, such as immunization and inflammation, and was always upregulated during tumorigenesis [20]. In colorectal cancer cells, miR-196a-5p targeted I*κ*B*α* to participate in epithelial-mesenchymal transition, invasion, and metastasis [21]. In STAD, Martins et al. suggested that miR-196a-5p was closely associated with immune-related pathways but the specific situation was not clearly identified [22]. Interestingly, Lario et al. elaborated that miR-196a-5p was downregulated in the precancerous lesions of gastric cancer [23]. The mechanism by which miR-196a-5p expression levels changed when precancerous lesions of gastric cancer were transformed into gastric cancer is worthy of further study.

As for miR-1-3p, increasing studies have focused on miR-1-3p in many cancers but only few have been conducted on STAD. For instance, the expression level of miR-1-3p was suppressed by a long noncoding RNA component of mitochondrial RNA-processing endoribonuclease, which induced the proliferation, migration, and invasion of non-small-cell lung cancer cells [24]. Additionally, miR-1-3p inhibited the proliferation of hepatocellular carcinoma cells by regulating SRY- (sex-determining region Y-) box 9 [25]. In gastric cancer, miR-1-3p negatively regulated stanniocalcin 2 expression, thereby inhibiting cell proliferation and invasion [26]. Through our study, we were the first to clarify the relationship between the expression level of miR-1-3p and clinical manifestations, such as TMN stage and invasion depth, in STAD. Further research on the role of miR-1-3p in STAD is required.

Serum miR-149-5p also showed an ability to diagnose STAD. Previous studies have confirmed that miR-149-5p is a tumor suppressor in STAD [27]. Researchers have focused on the miR-149 family and its target site in gastric cancer. Zhang et al. showed that miR-149-5p was taken up by the circular RNA, circNRIP1, and thereby caused tumorigenesis through the AKT1/mTOR pathway in STAD [28]. In addition, miR-149-5p was also considered a mediator of the progression of other cancers. For example, miR-149-5p played a role in the has-circ-0005615/miR149-5p/TNKS axis in colorectal cancer [29] and acted on the long noncoding RNA PART1/miR-149-5p/MAP 2K1 axis in hepatocellular carcinoma [30]. These results indicated that miR-149-5p greatly contributed to tumorigenesis. Therefore, it is understandable that miR-149-5p may act as a diagnostic biomarker for STAD.

The KEGG pathway analysis suggested that the neurotrophin signaling pathway was most relevant for the 4 identified miRNAs (Figure 6(d)). The study by Wei et al. similarly demonstrated the role of the neurotrophin signaling pathway in gastric cancer [31]. Four growth factors (NGF, BDNF, NT-3, and NT-4/5) and 2 types of receptors (Trk tyrosine kinase receptors and P75^NTR^) constitute the neurotrophin signaling pathway, which is associated with cancer stem cells [32]. Okugawa et al. reported that the BDNF/TrkB pathway may play an important role in gastric cancer progression [33]. The mechanism by which the neurotrophin signaling pathway is involved in STAD of gastric cancer shows great research prospects.

Although the conclusion is meaningful, the limitations of this study should not be ignored. First, our study only involved 28 initial miRNAs and left out many miRNAs associated with STAD. Additionally, the sample size of the screening phase was relatively small. Moreover, the expression levels of the 4 candidate miRNAs in the 130 STAD patients after surgery were not tested. It was still unclear whether the expression levels of the four miRNAs changed. Therefore, further studies are necessary.

In summary, a 4-miRNA panel that can be considered as a noninvasive biomarker for STAD diagnosis can be used to enhance STAD diagnosis. We further confirmed the relationship between miR-125b-5p, miR-196a-5p, miR-1-3p, and miR-149-5p and STAD, which provides an experimental basis for STAD research. Furthermore, the neurotrophin signaling pathway might play an important role in STAD progression.

## Figures and Tables

**Table 1:** Demographic and clinical manifestation of 246 participants (STAD and HCs).

	Screening phase (*n* = 32)			Training phase (*n* = 54)			Validation phase (*n* = 160)		
	STAD (%)	HC (%)		STAD (%)	HC (%)		STAD (%)	HC (%)	
Total number	20	12		30	24		80	80	
Age at diagnosis			*p* = 0.51			*p* = 0.33			*p* = 0.17
≤60	10 (50.0)	7 (58.3)		13 (43.3)	16 (66.7)		55 (68.8)	65 (81.2)	
>60	10 (50.0)	5 (41.7)		17 (56.7)	8 (33.3)		25 (31.2)	15 (18.8)	
Tumor diameter (mm)									
≤50	15 (75.0)			17 (56.7)			65 (81.2)		
>50	5 (25.0)			13 (43.3)			15 (18.8)		
Lymphatic metastasis									
Negative	8 (40.0)			11 (36.7)			20 (25.0)		
Positive	12 (60.0)			19 (63.3)			60 (75.0)		
TNM stage									
I-II	7 (35.0)			16 (53.3)			35 (43.8)		
III-IV	13 (65.0)			14 (46.7)			45 (56.2)		
Differentiation									
Poor	15 (75.0)			19 (63.3)			46 (57.5)		
Moderate-well	5 (25.0)			11 (36.7)			34 (42.5)		
Invasion depth									
T1-T2	8 (40.0)			14 (46.7)			33 (41.3)		
T3 - T4	12 (60.0)			16 (53.3)			47 (58.7)		

In all three phases, there was no significant difference between STAD patients and HCs based on age. The parameter values are presented as a number (percentage). Statistical contrast was exerted using the Kruskal-Wallis rank test.

**Table 2:** The association between the relative expression levels of the miRNAs in the serum miRNA and clinical manifestations (training and validation phases).

	hsa-miR-125b-5p		hsa-miR-196a-5p		hsa-miR-1-3p		hsa-miR-149-5p	
Tumor diameter (mm)		*p* = 0.90		*p* = 0.38		*p* = 0.60		*p* = 0.89
≤50	1.69 ± 0.77		1.70 ± 0.77		0.76 ± 0.32		0.79 ± 0.30	
>50	1.68 ± 0.67		1.50 ± 0.51		0.76 ± 0.24		0.82 ± 0.34	
Lymphatic metastasis		*p* = 0.43		*p* = 0.68		*p* = 0.66		*p* = 0.30
Negative	1.59 ± 0.59		1.72 ± 0.78		0.80 ± 0.33		0.86 ± 0.34	
Positive	1.73 ± 0.79		1.62 ± 0.70		0.74 ± 0.29		0.78 ± 0.30	
TNM stage		*p* = 0.015		*p* = 0.13		*p* < 0.001		*p* = 0.96
I-II	1.47 ± 0.55		1.55 ± 0.74		0.86 ± 0.29		0.81 ± 0.34	
III-IV	1.87 ± 0.83		1.73 ± 0.70		0.67 ± 0.28		0.80 ± 0.29	
Differentiation		*p* = 0.75		*p* = 0.39		*p* = 0.29		*p* = 0.83
Poor	1.73 ± 0.79		1.60 ± 0.70		0.74 ± 0.30		0.80 ± 0.28	
Moderate-well	1.63 ± 0.66		1.72 ± 0.75		0.79 ± 0.30		0.81 ± 0.35	
Invasion depth		*p* = 0.39		*p* = 0.28		*p* = 0.012		*p* = 0.56
T1-T2	1.60 ± 0.67		1.58 ± 0.75		0.83 ± 0.29		0.80 ± 0.34	
T3-T4	1.75 ± 0.79		1.70 ± 0.69		0.71 ± 0.29		0.81 ± 0.29	

Parameter values are presented as the mean ± SD. Statistical analysis was conducted using the Wilcoxon-Mann–Whitney test.

## Data Availability

Date are available if necessary.

## Supplementary Materials

Supplementary 1 Materials Figure S1: the framework of the study. STAD: stomach adenocarcinoma; HCs: healthy controls.

Supplementary 2 Materials Table S1: the statistics difference of 28 miRNAs between 5 STAD pools and 3 HCs pools at the screening phase.
