# Supplementary figures and images for: Use of a Four-miRNA Panel as a Biomarker for the Diagnosis of Stomach Adenocarcinoma
Source: Dis Markers. 2020 Nov 7;2020:8880937. doi: 10.1155/2020/8880937 (PMC7670587; doi:10.1155/2020/8880937)

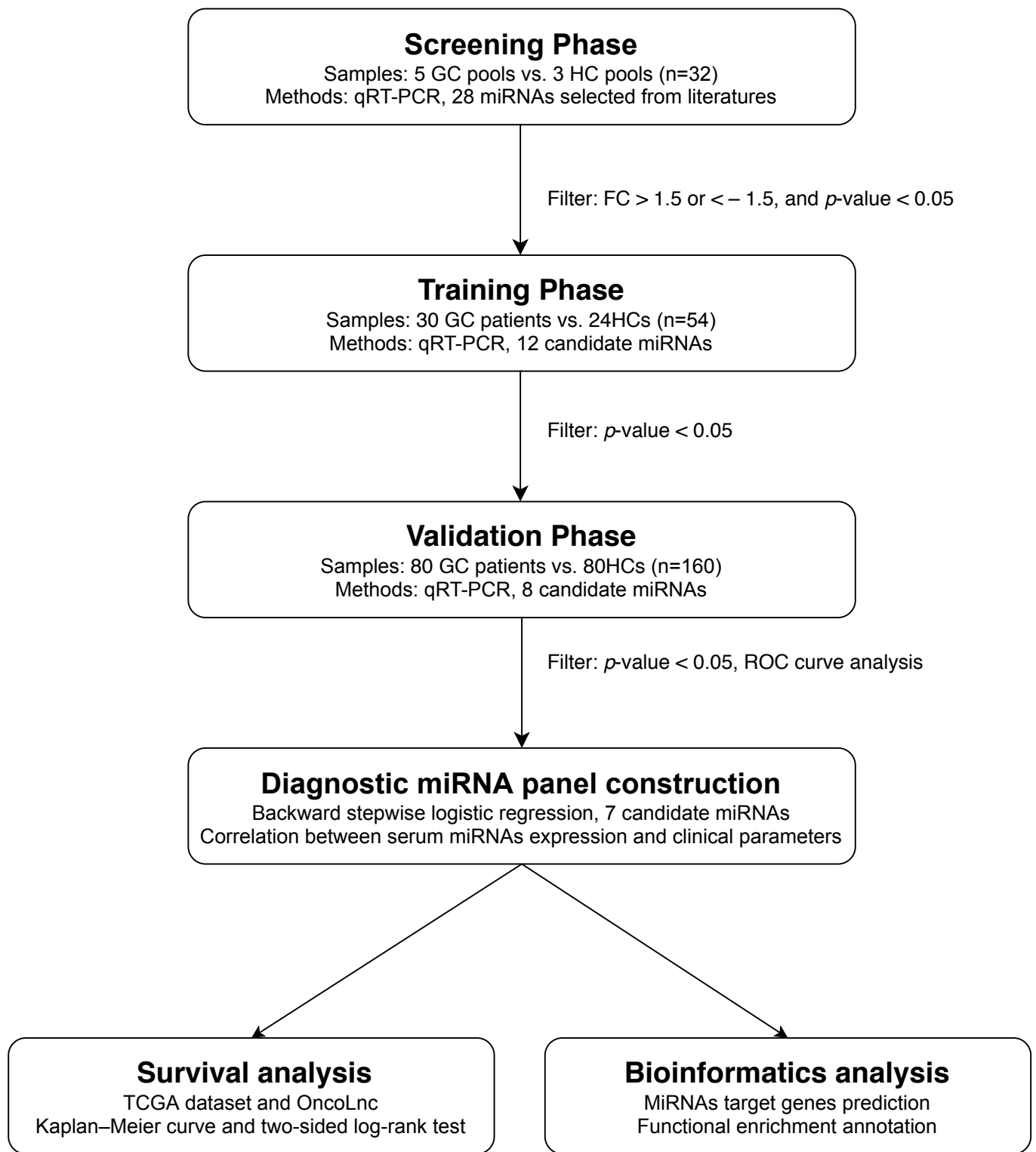

Supplement: Supplementary 1 — Materials Figure S1: the framework of the study. STAD: stomach adenocarcinoma; HCs: healthy controls. [file 8880937.f1.pdf]
