# Supplementary material for: Use of a Four-miRNA Panel as a Biomarker for the Diagnosis of Stomach Adenocarcinoma
Source: Dis Markers. 2020 Nov 7;2020:8880937. doi: 10.1155/2020/8880937 (PMC7670587; doi:10.1155/2020/8880937)
Supplement: Supplementary 2 — Materials Table S1: the statistics difference of 28 miRNAs between 5 STAD pools and 3 HCs pools at the screening phase. [file 8880937.f2.docx]

**Table S1** The statistics difference of 28 miRNAs between 5 STAD pools and 3 HCs pools at the screening phase.

| **ID** | **logFC** | **AveExpr** | **t** | **P.Value** | **adj.P.Val** | **B** |
| --- | --- | --- | --- | --- | --- | --- |
| **hsa-miR-196a-5p** | **2.351333** | **1.469583** | **10.84626** | **5.09E-07** | **4.76E-06** | **6.784851** |
| **hsa-miR-125b-5p** | **2.345556** | **1.465972** | **12.0548** | **1.82E-07** | **4.27E-06** | **7.838199** |
| **hsa-miR-9-5p** | **2.152222** | **1.345139** | **8.756996** | **3.89E-06** | **1.51E-05** | **4.678776** |
| **hsa-miR-182-5p** | **1.880222** | **1.175139** | **8.809213** | **3.68E-06** | **1.51E-05** | **4.736481** |
| **hsa-miR-124-3p** | **1.773333** | **1.108333** | **6.435683** | **6.10E-05** | **0.000155** | **1.795564** |
| hsa-miR-200a-3p | 1.428667 | 0.892917 | 3.973142 | 0.002409 | 0.004496 | -2.04331 |
| hsa-miR-195-5p | 1.248889 | 0.780556 | 4.968602 | 0.000492 | 0.001148 | -0.3928 |
| hsa-miR-199a-3p | 1.199222 | 0.749514 | 3.386394 | 0.006508 | 0.010719 | -3.06222 |
| hsa-miR-92b-3p | 1.158667 | 0.724167 | 4.637766 | 0.000822 | 0.001643 | -0.92773 |
| hsa-miR-181a-5p | 0.787778 | 0.492361 | 1.322041 | 0.214337 | 0.25006 | -6.39307 |
| hsa-miR-135b-5p | 0.630444 | 0.394028 | 2.542925 | 0.028304 | 0.037739 | -4.53266 |
| hsa-miR-129-5p | 0.495333 | 0.309583 | 2.012736 | 0.070603 | 0.085951 | -5.40864 |
| hsa-miR-574-3p | 0.284556 | 0.177847 | 0.68825 | 0.506273 | 0.556259 | -7.02137 |
| hsa-miR-1292-5p | 0.194222 | 0.121389 | 0.537013 | 0.602494 | 0.624809 | -7.11884 |
| hsa-miR-490-3p | 0.141556 | 0.088472 | 0.378282 | 0.712786 | 0.712786 | -7.19645 |
| hsa-miR-497-5p | -0.32267 | -0.20167 | -0.67139 | 0.516526 | 0.556259 | -7.03334 |
| hsa-miR-551b-3p | -0.67067 | -0.41917 | -2.16935 | 0.054073 | 0.06882 | -5.15737 |
| hsa-miR-202-3p | -0.74156 | -0.46347 | -2.55063 | 0.027926 | 0.037739 | -4.51949 |
| hsa-miR-383-5p | -0.84311 | -0.52694 | -2.68498 | 0.02208 | 0.034347 | -4.28853 |
| hsa-miR-140-5p | -1.05356 | -0.65847 | -2.62914 | 0.024346 | 0.035878 | -4.38481 |
| hsa-miR-155-5p | -1.37 | -0.85625 | -4.69346 | 0.000753 | 0.001621 | -0.83667 |
| **hsa-miR-224-5p** | **-1.62344** | **-1.01465** | **-3.66686** | **0.004029** | **0.00705** | **-2.57238** |
| **hsa-miR-100-5p** | **-1.698** | **-1.06125** | **-8.55166** | **4.85E-06** | **1.51E-05** | **4.449077** |
| **hsa-miR-105-5p** | **-2.08578** | **-1.30361** | **-8.12468** | **7.76E-06** | **2.17E-05** | **3.956773** |
| **hsa-miR-21-5p** | **-2.14156** | **-1.33847** | **-9.07725** | **2.78E-06** | **1.51E-05** | **5.028273** |
| **hsa-miR-143-3p** | **-2.27211** | **-1.42007** | **-8.89778** | **3.35E-06** | **1.51E-05** | **4.833708** |
| **hsa-miR-149-5p** | **-2.57222** | **-1.60764** | **-11.4361** | **3.05E-07** | **4.27E-06** | **7.312333** |
| **hsa-miR-1-3p** | **-2.64867** | **-1.65542** | **-8.57467** | **4.73E-06** | **1.51E-05** | **4.47504** |
